# Biochemical markers for assessment of calcium economy and bone metabolism: application in clinical trials from pharmaceutical agents to nutritional products

**DOI:** 10.1017/S0954422414000183

**Published:** 2014-12

**Authors:** Jean-Philippe Bonjour, Wendy Kohrt, Régis Levasseur, Michelle Warren, Susan Whiting, Marius Kraenzlin

**Affiliations:** 1 Division of Bone Disease, University Hospitals and Faculty of Medicine, Geneva, Switzerland; 2 Division of Geriatric Medicine, University of Colorado, Denver, CO, USA; 3 Service de Rhumatologie, CHU Angers, Angers, France; 4 Department of Obstetrics and Gynecology and Medicine, Columbia University Medical Center, New York, NY, USA; 5 College of Pharmacy and Nutrition, University of Saskatchewan, Saskatoon, Canada; 6 Division of Endocrinology, Diabetes and Metabolism, University Hospital, Bale, Switzerland

**Keywords:** Bone-related hormones, Bone turnover markers, Clinical nutrition trials

## Abstract

Nutrition plays an important role in osteoporosis prevention and treatment. Substantial progress in both laboratory analyses and clinical use of biochemical markers has modified the strategy of anti-osteoporotic drug development. The present review examines the use of biochemical markers in clinical research aimed at characterising the influence of foods or nutrients on bone metabolism. The two types of markers are: (i) specific hormonal factors related to bone; and (ii) bone turnover markers (BTM) that reflect bone cell metabolism. Of the former, vitamin D metabolites, parathyroid hormone, and insulin-like growth factor-I indicate responses to variations in the supply of bone-related nutrients, such as vitamin D, Ca, inorganic phosphate and protein. Thus modification in bone remodelling, the key process upon which both pharmaceutical agents and nutrients exert their anti-catabolic or anabolic actions, is revealed. Circulating BTM reflect either osteoclastic resorption or osteoblastic formation. Intervention with pharmacological agents showed that early changes in BTM predicted bone loss and subsequent osteoporotic fracture risk. New trials have documented the influence of nutrition on bone-tropic hormonal factors and BTM in adults, including situations of body-weight change, such as anorexia nervosa, and weight loss by obese subjects. In osteoporosis-prevention studies involving dietary manipulation, randomised cross-over trials are best suited to evaluate influences on bone metabolism, and insight into effects on bone metabolism may be gained within a relatively short time when biochemical markers are monitored.

## Introduction

The aim of the present report is to review how biochemical markers can be used to evaluate the potential benefit of foods or nutrients on adult bone health and in primary and secondary osteoporosis prevention. Nutrition and physical activity are key behavioural determinants of the development and progression of several chronic diseases, including osteoporosis. In primary prevention, nutrition combined with moderate physical activity plays an important role in attenuating age-related bone loss. In secondary prevention, adequate nutrition is adjuvant to specific anti-osteoporotic medications, thus enabling their full therapeutic effects.

This review focuses on the clinical application of biochemical analyses that can respond to nutritional interventions aimed at either preserving bone integrity or attenuating bone loss in adults.

Effects of foods and nutrients on Ca and inorganic phosphate (Pi) economy^(^ ^1^ ^)^ can be measured using bone-related circulating hormonal markers, such as vitamin D metabolites^(^ ^2^ ^)^, parathyroid hormone (PTH)^(^ ^3^ ^,^ ^4^ ^)^ and insulin-like growth factor (IGF)-I^(^ ^5^ ^)^, as well as bone turnover markers (BTM) that reflect bone formation and resorption^(^ ^6^ ^–^ ^9^ ^)^.

New findings on BTM have improved how they are viewed in the design, follow-up and interpretation of clinical trial outcomes, particularly in postmenopausal women and the elderly^(^ ^8^ ^,^ ^9^ ^)^. Indeed, BTM changes can predict the later influence of pharmaceutical agents on bone mineral density (BMD)/content and fragility fractures^(^ ^6^ ^–^ ^9^ ^)^. This will be discussed in the context of bone health-related claims as they relate to evidence-based knowledge of nutritional benefits. Examples of clinical trials are presented that document how nutrients influence bone-trophic hormones and biochemical markers of bone remodelling. Finally, appropriate study designs using both biochemical markers and new tools for assessing bone structure and function, as well as biomechanical properties, will be discussed, taking into account distinct adult populations.

## Osteoporosis, a major health problem

Osteoporosis is a systemic skeletal disease characterised by low bone mass and micro-architectural deterioration of bone tissue with a consequent increase in bone fragility and susceptibility to fracture that worsens with ageing^(^ ^10^ ^)^. Osteoporosis causes more than 8·9 million fractures annually worldwide^(^ ^10^ ^)^. The lifetime risk for wrist, hip or vertebral fracture is estimated to be 30 to 40 % in developed countries. Epidemiological studies in both the USA and UK indicate that one in two women aged 50 years or older will have an osteoporotic fracture in their remaining life; for men it is one in five^(^ ^11^ ^)^. The most serious osteoporotic fracture, at the proximal femur, typically results when subjects having an intrinsic bone fragility caused by both reduced mineral density and impaired structural design have fallen. These fractures show an exponential age-related increase and have a severe detrimental effect on the health of elderly populations^(^ ^10^ ^,^ ^11^ ^)^. Fragility fractures in elderly individuals are the long-term pathological consequence of an imbalance between bone resorption and bone formation leading to bone loss with micro-structural alterations of both cortical and trabecular structures^(^ ^10^ ^,^ ^11^ ^)^.

## Therapeutic options and limitations

There are many efficacious pharmaceutical options that have been shown, in large and well-designed randomised controlled trials (RCT), to significantly reduce the risk of fracture in postmenopausal women and the elderly^(^ ^12^ ^,^ ^13^ ^)^. Nevertheless, insufficient compliance in terms of both adherence and persistence to drug prescription limits their effectiveness for the management of individual patients in the real-world conditions of medical practice^(^ ^14^ ^–^ ^17^ ^)^.

It is relevant to note that in the stepwise drug development phases, virtually all testing of major anti-osteoporotic medications relied on BTM measurement to assess efficacy.

## Evidence-based criteria in osteoporosis management

Clinical evidence arises from the analysis of various research approaches with the aim of proposing the most reliable recommendations for practical disease management. The RCT is at the top of the strength-of-evidence hierarchy. These RCT usually measure non-vertebral fractures, and require that thousands of patients be followed for at least 3 years to reach a sufficient statistical power. However, some well-designed RCT testing specific pharmaceutical agents in postmenopausal women have failed to demonstrate efficacy for preventing hip fracture^(^ ^12^ ^)^. The incidence of hip fracture depends not only on the drug efficacy for improving bone structure and metabolism, but also on extra-skeletal factors, mainly those related to the occurrence and circumstances of falling. This makes hip fracture a stochastic event, largely independent of bone-active medications. Furthermore, RCT for osteoporosis typically provide both treatment and placebo groups with sufficient Ca and vitamin D, which may have independent benefits on bone metabolism and attenuate the potential effect size on hip fracture reduction.

## Concept of evidence-based nutrition in bone health and osteoporosis

Clinical research related to the impact of foods and/or nutrients on bone metabolism and structure is hampered by the difficulty to reach a level of evidence^(^ ^18^ ^,^ ^19^ ^)^ similar to that required of clinical trials that test pharmaceutical drugs for osteoporosis management^(^ ^20^ ^–^ ^22^ ^)^. It is difficult to prove the efficacy of drugs for preventing fragility fracture using large RCT designed for this purpose (see below), but it is even more challenging to use the RCT approach to evaluate whether foods or nutrients can reduce the risk of fragility fracture^(^ ^18^ ^,^ ^19^ ^)^. There is a major difference in the level of strength evidence required for foods or nutrients as compared with drugs, to evaluate not only benefits but also risks^(^ ^23^ ^–^ ^26^ ^)^. As explicitly stated by Heaney & Blumberg, ‘…we cannot get RCT-level evidence from many, and perhaps most, nutritional questions…’^(^ ^25^ ^)^. Therapeutic decisions using anti-osteoporotic drugs require evidence based on high-quality RCT that have as their main outcome the reduction of fragility fractures^(^ ^20^ ^)^. Inferences are weakened when studies rely on surrogate endpoints such as BMD or BTM^(^ ^20^ ^)^. Testing the efficacy of a food or nutrient with the reduction of hip fracture risk as the main outcome will probably never be amenable to RCT because it would require that thousands of patients remain compliant to the intervention for at least 3 to 4 years^(^ ^18^ ^,^ ^19^ ^,^ ^23^ ^)^. Industry is unlikely to sponsor these large RCT because, in contrast to drugs, marketing the products is not dependent on the results of RCT. However, as compared with foods or nutrients, the risk:benefit ratio is much greater with drugs, as they may cause more severe adverse side effects^(^ ^18^ ^,^ ^19^ ^,^ ^24^ ^,^ ^25^ ^)^.

In the nutrition field, valuable information could be obtained from RCT that have BTM endpoints reflecting bone metabolism. The change in BTM can be detected at a shorter time than the time for assessing effects on fragility fracture rate. The validity of results obtained from short-term RCT is strengthened by preclinical studies carried out in reliable animal models of human osteoporosis^(^ ^27^ ^–^ ^31^ ^)^. The next sections will deal with the scientific basis underlying these concepts.

## Bone-related biomarkers: biochemical analyses and clinical application

Both hormonal factors and biomarkers of bone remodelling can be useful to assess the baseline status and the response to an intervention in the context of prevention or treatment of osteoporosis.

Bone mass can be estimated by measuring BMD, i.e. areal BMD (aBMD) using dual-energy X-ray absorptiometry (DXA). While low aBMD is an important determinant of fracture risk, its measurement does not capture all the risk factors for fracture^(^ ^32^ ^)^. In fact, up to half of patients with incident fractures have baseline spinal or hip aBMD assessed by DXA above the diagnostic threshold of osteoporosis defined as a T-score of − 2·5 sd or more below the average value of young healthy adults (i.e. WHO definition of osteoporosis)^(^ ^33^ ^,^ ^34^ ^)^. Using peripheral measurement devices, 82 % of postmenopausal women with fracture had T scores better than − 2·5^(^ ^35^ ^)^. Furthermore, there is increasing evidence that the decision to use pharmacological intervention for the prevention of fracture should be based on the fracture probability rather than only on the presence of osteoporosis as defined by aBMD T-score^(^ ^32^ ^)^. Therefore, in selecting patients for treatment, the risk of fracture in individual subjects is now calculated by the use of algorithms which include a number of recognised independent risk factors for fracture in addition to aBMD, such as age, sex, BMI, family history, past history of fracture, secondary causes of osteoporosis such as rheumatoid arthritis, use of medications such as glucocorticoids, smoking and excessive alcohol intake^(^ ^32^ ^)^. FRAX® (WHO Fracture Risk Assessment Tool) is such a fracture risk calculator for the next 10 years (www.shef.ac.uk/FRAX/).

### Bone-related circulating hormonal factors

#### Hormonal changes to insufficient supply of calcium, vitamin D and protein

The main consequence of vitamin D insufficiency, as expressed by a low circulating level of 25-hydroxyvitamin D (25(OH)D), is a decrease in the intestinal absorption of both Ca and Pi. The reduction in the supply of Ca from the intestinal tract leads to an increase in the production of PTH to, at least partially, compensate for hypocalcaemia by stimulating both tubular Ca reabsorption and osteoclastic bone resorption. The secondary hyperparathyroidism also contributes to the associated hypophosphataemia resulting from the reduced tubular Pi reabsorption.

When associated with a deficiency of protein, as expressed by a low serum level of the anabolic agent IGF-I, osteoblastic bone formation rate no longer is matched to osteoclastic bone resorption rate. Moreover, insufficient dietary protein, in concert with insufficient vitamin D status, results in low intestinal Ca absorption. At the kidney, the reabsorption of Pi and the synthesis of the active metabolite of vitamin D, 1,25-dihydroxyvitamin D, tend also to be depressed. Overall, the insufficient dietary supply of these bone-trophic nutrients leads to an uncoupling between the resorption and the formation rate of the bone mineralised organic matrix that favours resorption. Intermediate and long-term possible consequences are measurable bone loss, by DXA or by high-resolution peripheral computed tomography^(^ ^36^ ^)^ and increased risk of fragility fracture.

In the course of dietary interventions aimed at correcting these nutrient insufficiencies, reliable biochemical assays are available to monitor changes in serum levels of 25(OH)D, PTH and IGF-I. In order to link these hormonal changes to the response of bone metabolism, BTM can concurrently be measured during the dietary intervention trial (see below).

#### Biochemical determination of bone-trophic hormonal factors: parathyroid hormone

A recommendation has been made to establish reference ranges for serum PTH in vitamin D-replete healthy individuals^(^ ^37^ ^)^, and to measure PTH in postmenopausal osteoporotic women to exclude secondary osteoporosis due to primary hyperparathyroidism^(^ ^3^ ^,^ ^38^ ^)^. Further, secondary hyperparathyroidism resulting from inadequate Ca intake and/or vitamin D deficiency, leading to an increase in bone resorption, is thus an important determinant of hip fractures in the elderly^(^ ^39^ ^–^ ^41^ ^)^. It has been shown by several investigators that acute oral Ca load or longer-term Ca supplementation (i.e. fortified dairy products) have favourable effects on bone metabolism by decreasing PTH and bone resorption markers^(^ ^42^ ^,^ ^43^ ^)^.

#### Biochemical determination of bone-trophic hormonal factors: 25-hydroxyvitamin D

The measurement of serum 25(OH)D is the best assessment of vitamin D status. The level associated with optimal suppression of circulating PTH levels has been used to define criteria for vitamin D insufficiency, because of the inverse relationship between serum 25(OH)D and PTH. However, this relationship may be influenced by age, Ca intake, physical inactivity, renal function, ethnicity and other factors. Furthermore, the use of different assays for 25(OH)D and PTH^(^ ^44^ ^,^ ^45^ ^)^ may also influence the apparent threshold of 25(OH)D concentration at which secondary hyperparathyroidism occurs. Conservatively, a 25(OH)D level of 50 nmol/l (20 ng/ml) is considered as vitamin D sufficient, representing a level above which there is no further suppression of PTH, not much further reduction in BTM, and not much further improvement of muscle function^(^ ^46^ ^)^. The Institute of Medicine also considers that serum levels of vitamin D above 50 nmol/l are the level that is needed for good bone health for practically all individuals, and set a recommended daily intake of 600 IU/d for children and adults and 800 IU for women and men 71 years or older based on this cut-off^(^ ^47^ ^)^. However, more recently, the clinical practice guidelines of the Endocrine Society defined vitamin D deficiency as 25(OH)D levels below 50 nmol/l (20 ng/ml) and vitamin D sufficiency as 25(OH)D levels greater than 75 nmol/l (30 ng/ml)^(^ ^48^ ^)^. In a cross-sectional analysis (National Health and Nutrition Survey (NHANES) 2003–2006) in the US population, maximum PTH suppression expected to occur when vitamin D is sufficient, corresponding to the zero slope between 25(OH)D and PTH, did not occur until a serum 25(OH)D level of approximately 100–125 nmol/l (40–50 ng/ml)^(^ ^49^ ^)^. Debate about the optimal vitamin D status continues^(^ ^49^ ^–^ ^51^ ^)^. There is evidence that Ca in combination with vitamin D supplementation reduces the risk of fracture^(^ ^52^ ^–^ ^55^ ^)^, bone loss^(^ ^56^ ^)^ and non-vertebral fracture risk^(^ ^52^ ^)^; these effects are more efficacious at 800 IU/d (20 μg/d) than 400 IU/d (10 μg/d).

#### Biochemical determination of bone-trophic hormonal factors: insulin-like growth factor-I

Measurement of serum IGF-I is an important tool with growth hormone (GH) not only in the diagnosis of various stature disorders^(^ ^57^ ^)^, but also as a nutritional marker, particularly of protein intake^(^ ^58^ ^,^ ^59^ ^)^. IGF-I and GH are regulators of bone homeostasis and are important for the acquisition of bone mass during adolescence, for the achievement of peak bone mass and for the maintenance of skeletal architecture during adult life^(^ ^60^ ^,^ ^61^ ^)^. Although GH may act directly on skeletal cells, most of its effects are mediated by IGF-I, which is present in the systemic circulation. Systemic IGF-I is synthesised in the liver under the influence of GH. IGF-I is also produced by a variety of cell types and acts in a paracrine fashion in many tissues. Availability of IGF-I is regulated by IGF binding proteins (IGFBP). Serum IGF-I levels are associated with bone mass. IGF-I levels peak at puberty when bone acquisition is maximal but levels of GH, IGF-I, and some IGFBP that regulate IGF activity decrease with age^(^ ^61^ ^–^ ^63^ ^)^. This decline in GH and IGF-I secretion has been correlated with bone loss in postmenopausal women. A relationship between IGF-I levels and fracture risk has also been reported^(^ ^64^ ^,^ ^65^ ^)^. However, it is not entirely clear how IGF-I is involved in the pathogenesis of osteoporosis, as serum IGF-I levels are strongly influenced by catabolic states, undernutrition and advanced age, all factors that increase the risk for fracture.

Dietary proteins influence both the production and action of IGF-I. Protein restriction is associated with reduced IGF-I levels by inducing a resistance to the GH action in the liver, and by an increased IGF-I metabolism. Decreased IGF-I levels are observed in states of undernutrition (i.e. also in anorexia nervosa). On the other hand, increased protein intake can prevent the decrease in IGF-I in hypoenergetic states. Experimental and clinical observational data suggest that IGF-I plays an important role in the pathophysiology of osteoporosis, osteoporotic fractures and its complications. Correcting altered IGF-I in the elderly by protein replenishment may favourably influence not only bone mass, but also muscle mass and strength that are important determinants for the risk of falls. The changes of IGF-I levels in nutrition-related changes in body weight are described below.

### Bone turnover markers

#### Importance of bone remodelling in osteoporosis

**Fig. 1:**
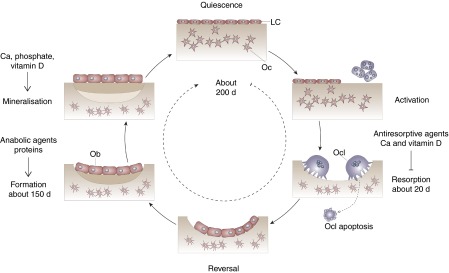
Bone remodelling cycle. Bone turnover follows a sequence of events that includes activation, recruitment of osteoclasts (Ocl) to begin resorption, degradation and removal of bone, reversal, and formation of new bone by osteoblasts (Ob). After this phase a quiescent or resting period occurs. LC, lining cell; Oc, osteocyte.

Bone remodelling is an important process that continuously renews the cortical and trabecular envelopes throughout life. It repairs micro-damage, maintains mineral homeostasis and ensures mechanical competence by modifying the micro-architecture. Bone remodelling is regulated by a variety of systemic and local factors as well as by nutritional factors (Fig. 1). Bone remodelling follows a sequence of events that includes activation, recruitment of osteoclasts to begin resorption, degradation and removal of bone, reversal, and formation of new bone by osteoblasts (Fig. 1). After this phase, a quiescent or resting period occurs. Remodelling begins with release of cytokines from osteoblast precursors, leading to recruitment of osteoclast precursors, which differentiate in multinucleated osteoclasts and subsequently attach to the bone surface. Resorption of the skeletal matrix releases growth factors such as IGF-I and transforming growth factor-β, which recruit lining cells and early osteoblast precursors that eventually form new bone (Fig. 1). Osteoblasts that do not undergo apoptosis remain on the bone surface as lining cells or become entrapped as osteocytes into the new bone matrix. These osteocytes respond to gravitational forces and can signal to lining cells to initiate remodelling. Antiresorptive and anabolic agents influence bone structure by affecting remodelling. Likewise, supply in Ca, vitamin D and protein influences bone remodelling. The presence of Ca and vitamin D inhibits bone resorption, and protein stimulates bone formation (Fig. 1). Ca, Pi and vitamin D are necessary for adequate mineralisation of the newly formed bone matrix (Fig. 1).

In young adulthood, the amount of bone removed by the osteoclasts is equal to the amount of bone formed by the osteoblasts, so that bone mass is maintained. After the menopause and with ageing in both sexes, there is a progressive bone loss due to an imbalance between bone resorption and formation.

There is a substantial body of evidence that accelerated bone turnover is associated with a higher risk of fracture, independent of other parameters such as age or BMD (see below). BTM are not included in the fracture risk calculator (FRAX®), mainly for the reason that their relationships with other established risk factors included in the risk calculator are not clarified and because different markers and different methodologies for their assessment have been used across studies. This has led to the recommendation for the standardisation of BTM measurements in future studies and it is suggested that serum carboxy terminal telopeptide of collagen type I (s-CTX) is used as the reference bone resorption marker and serum procollagen type I N-terminal propeptide (sPINP) as the reference bone formation marker, measured by standardised assays^(^ ^66^ ^)^.

### Bone markers reflecting bone formation and bone resorption

**Fig. 2:**
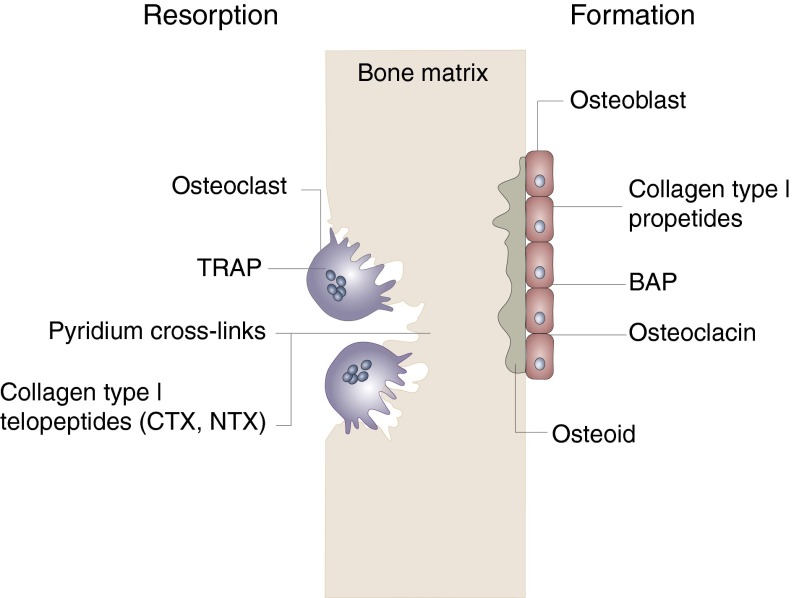
Diagram of the relationship between bone resorption or formation and bone turnover markers. On the resorption side, tartrate-resistant acid phosphatase (TRAP) reflects osteoclast number and activity, whereas pyridium cross-links, carboxy terminal telopeptide (CTX) and N-terminal telopeptide (NTX) are markers of bone matrix resorption. On the formation side, collagen type I propeptides, such as procollagen type I N-terminal propeptide (PINP), bone alkaline phosphatase (BAP) and osteocalcin reflect mainly osteoblastic bone formation. Osteoid corresponds to the non-mineralised bone matrix.

The BTM can be divided into two groups: markers of resorption and markers of formation (Fig. 2). The principal, specific markers of bone formation, measured in serum by immunoassays, are bone alkaline phosphatase (BAP), osteocalcin (OC) and PINP. Most assays are now adapted to automated platforms^(^ ^7^ ^)^ (Fig. 2). Markers of bone resorption are the pyridinium cross-links (pyridinoline and deoxypyridinoline) and their associated peptides (telopeptides), released during collagen breakdown^(^ ^6^ ^)^. Markers of osteoclast number include tartrate-resistant acid phosphatase isoenzyme 5b (TRAP 5b), the osteoclast-specific isoform of total TRAP, and, potentially, cathepsin K (Fig. 2). These enzymes are expressed and released by osteoclasts^(^ ^67^ ^)^. TRAP 5b is the most commonly used marker of osteoclast number.

TRAP 5b is predominantly expressed by osteoclasts, and immunoassays to detect this isoform have been developed^(^ ^68^ ^–^ ^70^ ^)^. In absolute terms, the response of TRAP 5b to antiresorptive therapy is not as great as the response of s-CTX, but within-subject variability is significantly lower. TRAP 5b has a lower diurnal variability than s-CTX and lower acute effect of feeding, possibly due to a longer circulating half-life, making it less susceptible to short-term influences^(^ ^71^ ^)^. Furthermore, TRAP 5b is not cleared by the kidney, and thus does not accumulate in renal failure^(^ ^69^ ^,^ ^71^ ^)^.

#### Predictive value of early changes in bone markers in response to pharmaceutical agents on later effects on bone mineral density/content and fragility fractures: bone turnover markers and the rate of bone loss

There is evidence that bone turnover increases rapidly after menopause and persists for up to 40 years^(^ ^72^ ^,^ ^73^ ^)^. In postmenopausal women bone turnover rate accounts for >50 % of the variation in bone mass, whereas in premenopausal women it is 0–10 %^(^ ^74^ ^,^ ^75^ ^)^.

Several prospective studies have demonstrated correlations between levels of BTM and rates of bone loss assessed by serial measurement of aBMD or bone mineral content at different skeletal sites over a time period spanning 13 years^(^ ^76^ ^)^. These studies support an inverse relationship between BTM and aBMD. This inverse relationship becomes stronger with advancing age and the relationship is significantly stronger for resorption markers than for formation markers, due to the fact that the increase of bone resorption with ageing is greater than the increase in bone formation^(^ ^77^ ^)^. However, this inverse correlation between BTM and aBMD does not seem to apply to all skeletal sites. A significant correlation between the rate of turnover and rate of bone loss at the hip or radius has been demonstrated in postmenopausal women, but not for the bone loss at the spine^(^ ^78^ ^,^ ^79^ ^)^. Extra-skeletal calcification and/or compression fracture occurring with advancing age can overestimate aBMD measurements at the spine. There is also evidence indicating that bone resorption markers can detect ‘rapid losers’ (>3 % loss in aBMD per year) in a follow-up period of 2–12 years^(^ ^80^ ^–^ ^82^ ^)^. At the present time a single measurement of a biochemical marker cannot predict the absolute rate of bone loss in a single individual. However, increased BTM can be regarded as a risk factor for rapid bone loss.

#### Predictive value of early changes in bone markers in response to pharmaceutical agents on later effects on bone mineral density/content and fragility fractures: bone turnover markers and fracture risk

BTM may be useful in the assessment of fracture risk. Findings of prospective studies have shown that BTM of resorption predict fracture risk in postmenopausal women and in men (Table 1)^(^ ^74^ ^,^ ^83^ ^–^ ^89^ ^)^. In five prospective studies of postmenopausal women (Epidemiology of Osteoporosis (EPIDOS), Rotterdam, Os de Femmes de Lyon (OFELY), Hawaii Osteoporosis Study (HOS), and Malmö Osteoporosis Prospective Risk Assessment (OPRA)), there were significant associations of baseline levels of urinary or s-CTX, urinary free deoxypyridinoline, or serum TRAP 5b with fracture risk^(^ ^74^ ^,^ ^83^ ^–^ ^87^ ^)^ (Table 1). An increase of these markers above the premenopausal range was associated, after adjustment for aBMD, with a twofold increase in risk for hip, vertebral and non-vertebral fractures, over a follow-up period of 1.8 to 5 years. The results for the relationship between bone formation markers and fracture risk are not consistent. In the French cohort study of elderly women (EPIDOS), no significant relationship between OC or BAP and fracture risk could be demonstrated during a 2-year follow-up. In contrast, in younger healthy postmenopausal women (OFELY and HOS cohorts), a significant association was found between increased BAP serum levels and risk of vertebral as well as non-vertebral fracture^(^ ^74^ ^,^ ^83^ ^,^ ^84^ ^)^.

**Table 1:** Relationship between increases in bone resorption rate and fracture risk

	Study	Age (years)	Fracture	RR-BMD*	95 % CI	Marker	RR-marker†	95 % CI
Women	EPIDOS^(^ ^74^ ^)^	>75	Hip	2·8	1·6, 5·1	Urinary CTX	2·2	1·3, 3·6
	OFELY^(^ ^83^ ^)^	64 (mean)	All	2·8	1·4, 5·6	Urinary CTX	2·3	1·3, 4·1
						Serum CTX	2·1	1·1, 3·6
	HOS^(^ ^84^ ^)^	69 (mean)	All	1·6	1·2, 2·2	Urinary CTX	1·6	1·2, 2·0
	Rotterdam^(^ ^85^ ^)^	>75	All	1·3	0·6, 2·8	Urinary DPD	1·9	1·2, 3·8
	Malmö OPRA^(^ ^86^ ^)^	>75	All	2·2	1·5, 3·1	TRAP	2·2	1·2, 4·2
Men	DOES^(^ ^87^ ^)^	72 (mean)	All	1·8	1·4, 2·3	ICTP	1·4	1·0, 1·9
							2·8‡	1·4, 5·4

RR, relative risk; BMD, bone mineral density; EPIDOS, Epidemiology of Osteoporosis; CTX, carboxy terminal telopeptide of collagen type I; OFELY, Os de Femmes de Lyon; HOS, Hawaii Osteoporosis Study; DPD, free deoxypyridinoline; OPRA, Osteoporosis Prospective Risk Assessment; TRAP, tartrate-resistant acid phosphatase; DOES, Dubbo Osteoporosis Epidemiology Study; ICTP, C-terminal telopeptide generated by metalloproteinases.

* RR-BMD =  RR for fracture by 1 sd decrease in BMD.

† RR-marker =  RR for fracture by 1 sd increase in marker above the premenopausal normal range.

‡ For the highest quintile.

These results suggest that a combined approach, with both aBMD and indices of bone turnover, could improve fracture prediction in postmenopausal women. In fact, calculating the absolute risk of fracture by combining clinical risk factor (i.e. previous fracture), low aBMD and increased bone turnover (high CTX) resulted in a better hip fracture probability at 10 years than with low aBMD alone^(^ ^90^ ^)^. In elderly men high bone resorption was also associated with an increased risk of osteoporotic fracture^(^ ^87^ ^)^.

#### Bone turnover markers and treatment monitoring

**Fig. 3:**
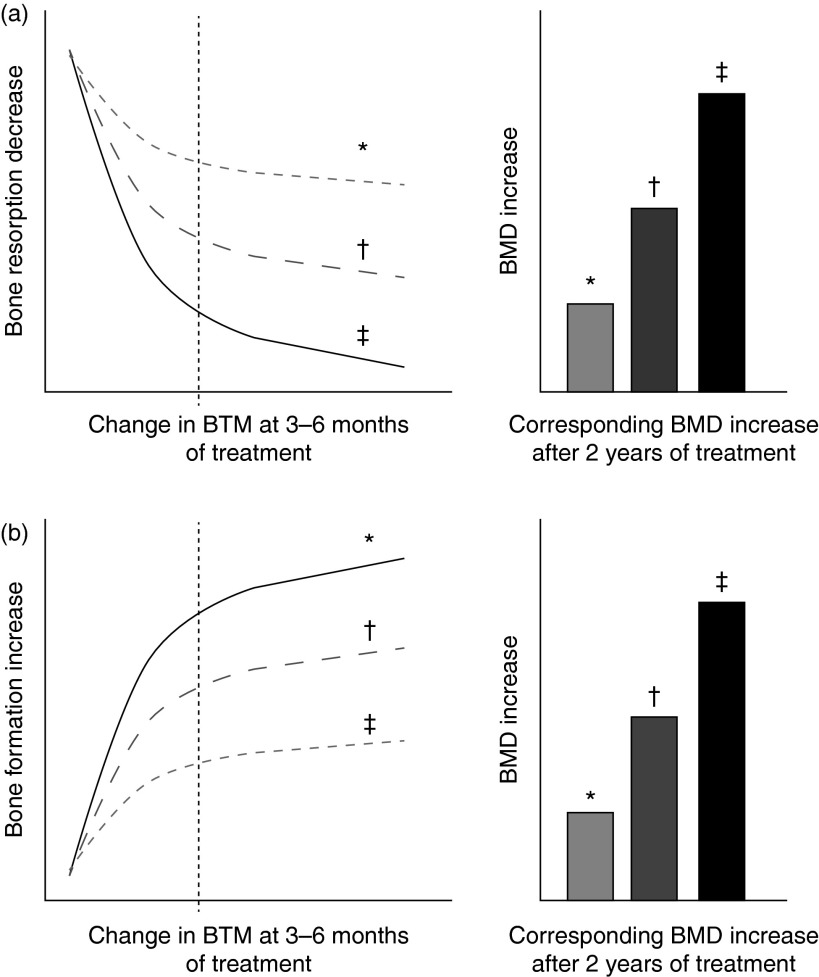
Schematic representation of changes in bone turnover markers (BTM) at 3–6 months and corresponding increase in bone mineral density (BMD) during treatment of osteoporosis. (a) Bone antiresorptive treatment (i.e. bisphosphonate): a more pronounced decrease in bone resorption marker at 3–6 months is associated with larger increases in BMD. (b) Bone formation-stimulating treatment (i.e. recombinant parathyroid hormone): a greater increase in bone formation marker is associated with a larger increase in BMD. * Mild response in changes in BTM and BMD. † Moderate response in changes in BTM and BMD. ‡ Pronounced response in changes in BTM and BMD. The diagrams show the relationship between early changes (3–6 months) in BTM and changes in BMD after 2 years of either bone resorption inhibition (a) or bone formation stimulation (b). Fig. 3(a) is based on quantitative data obtained by antiresorptive treatment with alendronate, documenting tertile changes at 6 months of the bone resorption marker urinary N-terminal telopeptide (u-NTX) and the changes in BMD as assessed by dual-energy X-ray absorptiometry (DXA) in total hip, trochanter and spine at 3 years in postmenopausal women^(^ ^98^ ^)^. Fig. 3(b) is based on quantitative data obtained by anabolic treatment with parathyroid hormone documenting tertile changes at 3 months of the bone formation marker N-terminal procollagen I propeptide (PINP) and the change in areal and volumetric BMD at 12 months, as assessed in the spine by DXA and quantitative computed tomography in postmenopausal women^(^ ^98^ ^,^ ^113^ ^)^.

The main domain for the clinical use of BTM is the monitoring of osteoporosis therapy. The ultimate goal in treating patients with osteoporosis is to reduce their fracture risk. However, the short-term incidence of osteoporotic fractures is low, and the absence of fracture during treatment does not necessarily mean that the treatment is effective. Consequently, serial measurements of changes in aBMD as a surrogate marker of therapeutic efficacy are currently the standard approach to monitor osteoporosis therapy. Yet, changes in aBMD occur slowly and therapeutic effects are usually not detectable before 1–2 or more years of treatment^(^ ^80^ ^,^ ^91^ ^–^ ^94^ ^)^. In contrast, BTM levels change much faster than aBMD in response to therapeutic interventions^(^ ^80^ ^)^. Antiresorptive treatment generates a rapid decrease in bone resorption markers after only 2–4 weeks, reaching a plateau after 3 to 6 months of treatment^(^ ^80^ ^,^ ^95^ ^)^ (Fig. 3(a)). The decrease in bone formation markers, reflecting the physiological coupling of bone formation to bone resorption, is delayed and reaches a plateau after 6–12 months. The magnitude of the decreases varies according to the type and dose of the drug and the marker used to assess the effect (Table 2).

**Table 2:** Changes from baseline in serum tartrate-resistant acid phosphatase (s-TRAP), carboxy terminal telopeptide of collagen type I (s-CTX) and procollagen type I N-terminal propetide (s-PINP) in response to antiresorptive and bone formation-stimulating treatments*

	Change from baseline (%)
Compound	Dose and route of administration	s-TRAP	s-CTX	s-PINP
Ca + vitamin D	500–1000 mg +10–20 μg/d (p.o.)	− 20 to − 25	− 20 to − 30	− 10 to − 20
Alendronate	70 mg/week (p.o.)	− 30 to − 40	− 70 to − 80	− 60 to − 70
Risedronate	35 mg/week (p.o.)		− 50 to − 60	− 40 to − 50
Ibandronate	150 mg/month (p.o.)			
	3 mg/3 months (i.v.)		− 60 to − 80	− 60 to − 70
Zoledronate	5 mg/year (i.v.)		− 50 to − 60	− 50 to − 60
Raloxifene	60 mg/d (p.o.)	− 10 to − 15	− 20 to − 50	− 30 to − 40
Denosumab	60 mg/6 months (s.c.)		− 70 to − 90	− 60 to − 70
PTH 1–34 (teriparatide)	20 μg/d (s.c.)		Up to 80	Up to 200
PTH 1–84	100 μg/d (s.c.)		10–100	90–150

p.o., Oral; i.v., intravenous; s.c., subcutaneous; PTH, parathyroid hormone.

* Data have been adapted from the references Naylor & Eastell^(^ ^8^ ^)^, Henriksen *et al.* ^(^ ^67^ ^)^ and Bonjour *et al.* ^(^ ^127^ ^)^.

Evidence from interventional drug trials, where the placebo groups received Ca and vitamin D, as well as in nutritional interventions with fortified food, shows a reduction in bone resorption markers of 20–30 % (as outlined in more detail in the ‘Study designs to test the effects of food products on bone metabolism’ section). The decrease in BTM during antiresorptive treatment is inversely related to the subsequent increase in aBMD, predominantly at the lumbar spine^(^ ^80^ ^,^ ^81^ ^)^. Several studies of postmenopausal women treated with antiresorptive agents (hormone replacement therapy, raloxifen, bisphosphonates) have indicated that the degree of short-term reduction in BTM (after 3 to 6 months) correlates with the observed long-term increase in aBMD after 1 to 3 years of treatment^(^ ^79^ ^,^ ^81^ ^,^ ^95^ ^–^ ^99^ ^)^. Reduction in fracture risk is only partially explained by the documented changes in aBMD (4–28 %)^(^ ^91^ ^,^ ^100^ ^–^ ^104^ ^)^. There is also some evidence that even a modest decrease in bone mass is nevertheless associated with some fracture risk reduction^(^ ^105^ ^)^. There is thus substantial evidence that bone mass or changes thereof does not explain satisfactorily either the skeletal fragility of osteoporosis or the effects of bone active agents^(^ ^106^ ^)^. It is therefore possible that changes in other determinants of bone strength, including the rate of bone turnover, may be more predictive of anti-fracture efficacy than changes in aBMD. In fact, several studies confirmed that short-term reductions in bone turnover were associated with a reduction in vertebral and/or non-vertebral fracture risk in women treated with hormone replacement therapy, raloxifene, risedronate and alendronate^(^ ^88^ ^,^ ^102^ ^,^ ^107^ ^–^ ^109^ ^)^.

In contrast to antiresorptive agents, PTH fragments administered intermittently in low doses increase bone remodelling, stimulating bone formation preferentially over bone resorption, and resulting in net gain of bone and reducing fracture risk^(^ ^110^ ^–^ ^112^ ^)^. Increases in BTM indicate a skeletal response to PTH. There is evidence that early measurement of bone formation markers (OC, BAP and particularly PINP) correlates positively with the subsequent BMD response to PTH (Fig. 3(b))^(^ ^113^ ^–^ ^116^ ^)^. These data suggest that serial BTM measurements could become useful in identifying skeletal responders to an anabolic therapy with PTH. Based on results from clinical studies and improved technical performance on automated platforms, several national and international guidelines recommend these markers in the follow-up of patients treated for postmenopausal osteoporosis^(^ ^32^ ^,^ ^117^ ^,^ ^118^ ^)^.

### Usefulness of biomarkers of bone turnover in clinical nutrition research

In the process of generating scientific support for claims on foods, BTM were deemed as not sufficiently indicative of bone health to provide evidence for enhanced function or reduced osteoporosis risk^(^ ^29^ ^)^. However, over the last 10 years there have been improvements in analytical performance and new knowledge of how biochemical markers can be of great utility in osteoporosis management. Because BTM measurement can assess response to anti-osteoporotic medications, the time has come to use BTM levels to reflect bone formation and resorption in response to foods and nutrients.

## Influence of foods or nutrients on bone turnover markers

Several studies have examined the response of BTM levels and related hormonal factors to varying food or nutrient consumption in postmenopausal women or in elderly individuals. These studies illustrate how BTM can be important in establishing food and nutrient health claims.

### Postmenopausal women

**Fig. 4:**
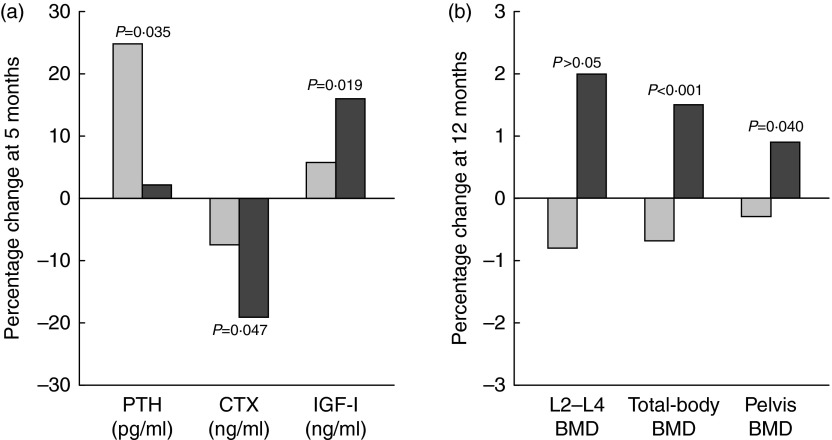
Changes (%) in serum biochemical indexes at 5 months (a) and in bone mineral density (BMD) at 12 months (b) in response to fortified dairy products (▓) for 12 months as compared with controls (░) in postmenopausal women. Data are adapted from Manios *et al.* ^(^ ^119^ ^)^. PTH, parathyroid hormone; CTX, carboxy terminal telopeptide of collagen type I; IGF-I, insulin-like growth factor-I. *P*= Probability level for treatment ×  time interaction effect as compared with the control group.

In a 12-month intervention involving postmenopausal women (mean age 60–62 years), the consumption of Ca- and vitamin D-fortified milk and yogurt favourably influenced vitamin D status and bone metabolism, as expressed by changes in serum 25(OH)D, PTH, CTX and IGF-I, as compared with a control group maintaining their usual diet or a group receiving equivalent Ca as lactate–gluconate and bicarbonate salts^(^ ^119^ ^)^ (Fig. 4). A prospective cross-over trial in postmenopausal women (mean age 59 years) found that supplementation with non-fortified milk for 6 weeks induced a decrease in serum PTH and BTM, including CTX, and a non-significant increase in IGF-I^(^ ^120^ ^)^. In a randomised study of postmenopausal women (mean age 57 years), consumption of a vitamin D- and Ca-fortified soft white cheese significantly reduced the sensitive biochemical marker of bone resorption TRAP 5b and increased serum IGF-I levels^(^ ^121^ ^)^.

### Elderly individuals

Favourable effects of milk, fortified or not with vitamin D or Ca, on bone turnover in elderly individuals have been documented in several intervention studies^(^ ^42^ ^,^ ^120^ ^,^ ^122^ ^–^ ^124^ ^)^.

Interventions with dairy products such as yogurts^(^ ^125^ ^,^ ^126^ ^)^ or soft white cheese^(^ ^126^ ^–^ ^128^ ^)^ have shown reductions in bone turnover. In institutionalised elderly individuals with vitamin D deficiency and secondary hyperparathyroidism, an 8-week consumption of vitamin D- and Ca-fortified yogurts saw increases in serum 25(OH)D, associated with a reduction in the BTM CTX and TRAP 5b, and a normalisation of previously high serum PTH^(^ ^126^ ^)^. Elderly patients hospitalised for an osteoporotic fracture of the proximal femur often present with signs of undernutrition, particularly protein malnutrition^(^ ^129^ ^)^. In a randomised study, protein repletion by the daily consumption of a 20 g casein-enriched food *v.* an isoenergetic non-protein supplement led to a significant elevation in serum IGF-I by the end of the 6-month dietary intervention^(^ ^58^ ^)^. The serum IGF-I response to 20 g/d protein repletion in patients with a recent hip fracture was observable by 7 d, with no further rise in serum IGF-I^(^ ^130^ ^)^. Thus, in hip fracture patients, the long-term effects of various protein preparations on IGF-I can be predicted from changes observed as early as 1 week following protein supplementation^(^ ^130^ ^)^.

## Bone turnover markers in clinical research on eating disorders

Several nutrition-related disorders can lead to bone loss and increased risk of osteoporotic fracture. Their pathophysiology and clinical expression in relation to the application of biochemical markers are relevant to the present review.

### Anorexia nervosa

In young women with anorexia nervosa, aBMD is reduced at several skeletal sites^(^ ^131^ ^)^. Body weight, but not oestrogen use, is a significant predictor of aBMD in these patients. Together with low endogenous oestrogen levels and Ca deficiency, a low protein intake very probably also contributes to the bone deficit observed in anorexia nervosa. However, low Ca intake has not been consistently reported and 25(OH)D serum levels are usually normal^(^ ^132^ ^)^. The BTM of bone formation, serum OC and BAP, are significantly reduced in adolescents whereas in adults with anorexia nervosa, the markers of bone resorption are elevated while those of formation are suppressed^(^ ^133^ ^,^ ^134^ ^)^. The serum level of IGF-I is low in anorexia nervosa^(^ ^135^ ^,^ ^136^ ^)^, an observation quite compatible with insufficient protein intake^(^ ^135^ ^)^ although low protein intake has not been consistently recorded. Nutritional rehabilitation reversed the uncoupling of bone remodelling^(^ ^137^ ^)^ and led to a significant increase in BTM of formation followed by increases in aBMD in as little as 3 months^(^ ^134^ ^)^. This reversibility occurs before oestrogen levels rise and menses return, suggesting that nutritional factors are of paramount importance^(^ ^132^ ^,^ ^134^ ^)^.

The serum level of IGF-I was reported to increase rapidly after the onset of intravenous hyperalimentation, followed by a progressive elevation in serum osteocalcin^(^ ^136^ ^)^. IGF-I level changes were dependent on variations in the nutritional state^(^ ^133^ ^)^. Increased GH levels accompanied by decreased IGF-I are seen in anorexia nervosa, suggesting an acquired resistance to GH that reversed with refeeding^(^ ^132^ ^)^. The administration of recombinant IGF-I alone has only a small bone-sparing effect^(^ ^138^ ^)^. This is not surprising, since the anabolic effects of IGI-I on bone formation are tempered by poor nutrition, particularly by insufficient protein intake^(^ ^139^ ^)^.

### Intensive exercise

In female athletes or ballet dancers, intensive exercise in the absence of appropriate nutrition can lead to hypothalamic dysfunction with delayed menarche and disruption of menstrual cyclicity, bone loss and increase risk of fracture^(^ ^140^ ^–^ ^142^ ^)^. Nutritional restriction, more common when leanness confers advantages for performance, can play an important role in the disturbance of the female reproductive system when combined with intense physical activity. Insufficient energy intake with respect to energy expenditure impairs the secretion of gonadotropin-releasing hormone and thereby leads to a state of hypo-oestrogenism. This hormonal disturbance is reversed by nutritional rehabilitation leading to an increase in both luteinising hormone and follicle-stimulating hormone. These changes occur well before oestrogen rises and menses return, suggesting the dominant effect of nutritional factors^(^ ^132^ ^)^. However, the relative contribution of insufficient protein intake with low IGF-I remains to be assessed since it is often associated with reduced energy intake. The consequence on bone metabolism of acute experimental energy restriction has been investigated in young exercising female volunteers^(^ ^143^ ^)^. This study indicated that severe reduction in energy availability rapidly induces a negative uncoupling between bone formation and bone resorption^(^ ^143^ ^)^. The resulting bone loss may become irreversible with persistence of such a negative uncoupled remodelling, explaining the increased risk of fragility fractures in anorexia nervosa^(^ ^132^ ^,^ ^144^ ^)^ as well as in certain athletes and ballet dancers^(^ ^140^ ^,^ ^142^ ^)^.

### Induced weight loss in overweight/obese subjects

Reduction in energy balance induced by energy restriction is often associated with bone loss that varies according to sex, age, ovarian function and initial fat mass (for a recent review, see Shapses & Sukumar^(^ ^145^ ^)^). The degree of bone loss also depends upon the kind of diet accompanying the energy restriction. Bone mineral loss during body-weight reduction may not be fully recovered with weight regained, at least in postmenopausal women^(^ ^146^ ^)^. Hence, it is important to understand the underlying pathophysiological mechanisms and thereby define the best strategies to prevent bone loss during energy restriction in overweight/obese individuals. Several studies have used bone health biomarkers, particularly with the objective to identify the key implicated nutrients.

Ca intake typically decreases with energy-restriction diets. In this condition, the reduced Ca intake is associated with elevation in both serum PTH and biochemical markers of bone remodelling^(^ ^147^ ^,^ ^148^ ^)^. Consumption of Ca salt supplements can suppress the rise in both serum PTH and BTM^(^ ^147^ ^,^ ^149^ ^–^ ^151^ ^)^.

Dairy products may prevent bone loss during energy restriction not only by providing Ca but also by supplying proteins. Weight-reducing diets that are high in dairy products can suppress the rise in bone turnover and prevent bone loss^(^ ^152^ ^,^ ^153^ ^)^. In postmenopausal women, a randomised 12-month controlled study showed that higher dietary protein intake (86 *v.* 60 g/d) during weight reduction increased serum IGF-I and attenuated loss of aBMD at several skeletal sites and volumetric BMD at the distal tibia^(^ ^154^ ^)^. The additional consumed proteins were from various food sources including dairy products, with a whey supplement providing 6 g protein/d^(^ ^154^ ^)^. Combining dairy foods with physical exercise^(^ ^155^ ^,^ ^156^ ^)^ might be appropriate to prevent bone loss during weight loss in overweight/obese human adults^(^ ^157^ ^)^. In a recent study, hypo-energetic diets higher in dairy Ca and protein combined with daily exercise favourably affected bone health biomarkers as follows: decrease in PTH; increase in bone formation markers (OC and PINP) with no change in bone resorption markers (CTX and N-terminal telopeptide (NTX)); increment in osteoprotegerin:receptor activator of NFκB ligand (RANKL) ratio^(^ ^157^ ^)^.

## Study designs to test the effects of food products on bone metabolism

In order to provide information on medical practice, the needed evidence-based criteria differ for foods and nutrients as compared with drugs. The development of drugs requires a very high level of rigour, more for safety concerns than for providing evidence of efficacy^(^ ^18^ ^,^ ^24^ ^,^ ^25^ ^)^. The present review essentially considers the rationale for using BTM in clinical research on foods or nutrients that influence bone mineral economy and/or metabolism.

### Issues with randomised controlled trials in osteoporosis

An RCT is the clinical study design that best permits strong causal inference between a tested drug or nutrient and a given outcome^(^ ^18^ ^,^ ^20^ ^)^. Previous reviews or position papers have emphasised the distinct differences in the evidence that can be obtained by using RCT in the context of drug testing, as compared with the development of nutrient requirement or dietary guidelines^(^ ^18^ ^,^ ^23^ ^,^ ^24^ ^,^ ^158^ ^,^ ^159^ ^)^. In the osteoporosis context, in order to demonstrate the efficacy of a new medication, regulatory agencies usually require RCT of 3 years’ duration, with fracture as the primary endpoint. In the nutrition field, all types of research are useful in setting dietary recommendations: observational studies, to document prevalence and generate hypotheses; preclinical experiments to identify physiological and biochemical pathways; qualitative studies to understand the human condition; and, finally, RCT, to establish a relationship of causality^(^ ^18^ ^,^ ^24^ ^,^ ^25^ ^,^ ^160^ ^)^.

### Ethical consideration in randomised controlled trials and sample size

The demonstration that a drug is efficacious for reducing the burden of fragility fractures necessitates the design of RCT with very large sample sizes. It is especially the case when, for ethical reasons, the new therapy is tested in so-called ‘active comparator trials’, i.e. when the recently developed medication is compared with an approved drug with convincing antifracture efficacy in patients at high risk of fragility fractures^(^ ^161^ ^)^. Moreover, a placebo-controlled trial can neither be considered ethically acceptable when the new drug is tested in patients at low risk of fractures. This study design requires a much larger number of subjects, as compared with previous RCT conducted in patients at high risk of fragility fractures^(^ ^161^ ^)^. Thus, proper design and execution of long-duration large RCT aimed at demonstrating the antifracture efficacy of a drug impose requirements that are difficult to meet^(^ ^158^ ^)^. This difficulty also encompasses both the narrow response due to the placebo effect and the non-random loss of enrolled subjects that convert a randomised trial into a cohort study^(^ ^158^ ^)^. It can be expected to be magnified in long-duration RCT aimed at testing the antifracture efficacy of foods or nutrients in osteoporotic patients^(^ ^18^ ^,^ ^158^ ^)^.

### Surrogate endpoints for antifracture efficacy of new drugs

Therefore, the development of surrogate endpoints for antifracture efficacy of new drugs has been considered not only for foods and nutrients^(^ ^158^ ^,^ ^159^ ^)^, but also for drugs^(^ ^161^ ^)^. Taking into account not only the large sample size but also the very long duration of conducting RCT with fragility fracture as the primary outcome, it is worth considering the measurement of fracture-related data that can be obtainable within a few weeks or months after the onset of the intervention. In clinical investigation, it is recognised that the early bone remodelling response is a good predictor of fragility fracture^(^ ^158^ ^,^ ^159^ ^)^. Effective pharmaceutical agents, whether acting via anticatabolic or anabolic mechanisms, very promptly influence bone remodelling, as biochemically expressed by measuring BTM changes^(^ ^158^ ^)^. These pharmoco-kinetic and -dynamic characteristics, that combine an early response to intervention with a reliable predictability of fragility fractures, make BTM tools better suited to assess the effects of foods or nutrients in clinical research on osteoporosis than the delayed changes in DXA-measured aBMD or bone mineral content. Furthermore, the effect size is usually larger with BTM after a few months than the changes in BMD/bone mineral content that can be observed after 1, or more frequently, 2 years of intervention.

### Short-duration randomised controlled trials in nutrition clinical research

In adulthood, the effects of foods on BTM can be assessed in appropriately designed RCT. Examples of such studies are described in the ‘Study designs to test the effects of food products on bone metabolism’ section. The short duration of these RCT limits the number of non-compliant participants. Furthermore, one may also expect that few subjects will be completely lost, i.e. without outcome measurement. This makes the ‘intention-to-treat’ data analysis more valuable for comparison with the ‘per protocol’ analysis that will only include treatment-adhering participants, and thereby provides an estimate of the effect size, some sort of ‘dose–response’, of the intervention.

A physiologically meaningful set of biochemical variables can be selected and assessed at several time points. For instance, when nutritional products are evaluated for their capacity to improve vitamin D status and bone metabolism, changes in serum 25(OH)D, PTH, bone formation and resorption markers should provide information on the dynamic interrelationships between these variables. Measuring the circulating level of IGF-I can assess the response to an increase in protein intake. These short-term RCT studies should provide not only qualitative information on the response but also quantitative data on both the magnitude and the pharmacokinetics of the effects.

### The advantage of cross-over design in nutritional investigation

A powerful methodology is the cross-over design, also called ‘*n* of 1 RCT’^(^ ^162^ ^,^ ^163^ ^)^. Herein there are two intervention periods, separated by a ‘washout’ period, the duration of which depends upon how long the effect of the intervention lingers after its discontinuation. For some anti-osteoporotic agents such as bisphosphonate compounds, the effect can last months or years. For foods and nutrients acting on bone, the effects may be ‘washed out’ much more rapidly, i.e. within a few days or weeks, with the exception of large doses of vitamin D. Within a trial, each participant acts as his or her own control, and permits comparison between and within groups^(^ ^164^ ^)^. This design eliminates any genetic influence and reduces bias associated with imbalance in known and unknown confounding variables, as compared with randomised parallel-group controlled trials enrolling two unrelated groups of subjects^(^ ^164^ ^)^.

### Comparative effectiveness research

An important recent trend in medical research is to apply ‘comparative effectiveness research’ (CER)^(^ ^165^ ^)^. CER is a marked departure from the efficacy standard that any new intervention should outperform placebo or randomised controls. There is particular emphasis placed on the leading question regarding clinical effectiveness of care. A CER study is aimed at determining the value of an intervention over another. It answers two relevant questions: (1) how effective is the intervention, if enrolled subjects follow it as prescribed?; (2) how likely is it that the enrolled participants follow it as prescribed?^(^ ^166^ ^)^. In a classically designed study, the answers to these two questions would be given by comparing the results computed by ‘intention to treat’ with those analysed ‘per protocol’. CER is a recommended priority for nutrition research^(^ ^165^ ^)^. Although CER will pose challenges for medical and nutritional industries, it will present numerous opportunities to improve the information base supporting nutritional products, especially when they are integrated into other medical interventions^(^ ^166^ ^)^. Recent CER nutrition studies have been reported on the bone effects of several vitamin D and Ca supplement regimens^(^ ^167^ ^,^ ^168^ ^)^.

## Conclusions

Osteoporosis is a major health problem for an increasing number of adults as they age. Nutrition plays a substantial role in the primary and secondary prevention of osteoporosis. Several nutrients exert reproducible beneficial effects on bone metabolism, reducing the acceleration of bone loss that occurs after the menopause and in the elderly. The supply in vitamin D, Ca, Pi and protein affects levels of bone-trophic hormones as well as resorption of the bony organic matrix and its subsequent formation and mineral deposition. Some foods and nutrients positively influence bone metabolism by either reducing bone resorption or increasing bone formation, or both. Measurement of several biochemical components in blood facilitate the delineation of the impact of nutrients and food factors on bone-trophic hormones such as vitamin D metabolites, PTH and IGF-I, and on BTM that reflect bone resorption and/or matrix formation and mineralisation. Cross-over randomised trials appear to be the optimal way to evaluate the influence of nutrients and food factors on bone metabolism. Using such a study design, which is considered near the top of the hierarchy of evidence for nutritional studies, biologically significant insight can be gained into the potential influence of food products on bone metabolism and osteoporosis prevention within a relatively short time.
